# Biophysics in Membrane of Cells

**DOI:** 10.3390/ijms241612708

**Published:** 2023-08-11

**Authors:** Songwen Tan, Wenhu Zhou

**Affiliations:** Xiangya School of Pharmaceutical Sciences, Central South University, Changsha 410013, China

The membrane of a cell, often compared to a dynamic city border, carries out an intricate dance of controlling entry and exit, guarding the valuable life processes occurring inside. Lipids and proteins are essential components of cell-surface membranes [1]. The dynamic remodeling of cell membranes and its underlying mechanisms have been extensively studied [2]. In this Special Issue, our focus shifted to understanding the subtleties of this border control—its structure, function, and more—through the lens of biophysics. This editorial aims to encapsulate key findings presented in the four original research papers and two comprehensive reviews featured in this issue, drawing common threads and envisioning future paths for this exciting field.

The four research papers presented in this Special Issue delved into the profound yet intricate relationship between biophysics and cellular functionality, with a particular emphasis on cryopreservation of cells and the lipid behavior in cell membranes.

The studies “Dimethylglycine Can Enhance the Cryopreservation of Red Blood Cells by Reducing Ice Formation and Oxidative Damage” [3] and “Tricine as a Novel Cryoprotectant with Osmotic Regulation, Ice Recrystallization Inhibition and Antioxidant Properties for Cryopreservation of Red Blood Cells” [4] have shed light on the evolving domain of cryoprotectants—a field bursting with potential for groundbreaking discoveries. Both dimethylglycine and tricine have emerged as potent candidates in bolstering the efficacy of Red Blood Cells cryopreservation. Remarkably, dimethylglycine has boosted recovery rates from a meager 11.55% to a commendable 72.15%. Meanwhile, tricine has carved a niche for itself as a formidable osmotic balancer and antioxidant. These advancements underscore the pivotal role that the identification of novel, potent, and benign cryoprotectants could play in the realm of blood transfusion treatments. Moreover, a plethora of chemical strategies for cryopreservation have been unveiled [5], particularly by Professor Gibson’s team, emphasizing ice recrystallization inhibition [6], ice-nucleating agents [7], and other intricate mechanisms.

Delving into the microcosm of lipid rafts, the studies “Lipids in Mitochondrial Macroautophagy: Phase Behavior of Bilayers Containing Cardiolipin and Ceramide” [8] and “Effect of Lipid Raft Disruptors on Cell Membrane Fluidity Studied by Fluorescence Spectroscopy” [9] examined the roles of specific lipids in the context of membrane function. From the molecular interactions between cardiolipin, ceramide, and autophagy proteins to the effects of raft disruptors on membrane fluidity, these studies illuminated the delicate balance within lipid rafts. An intriguing aspect that needs further exploration is the correlation between membrane fluidity and physiological functionality, potentially opening avenues for targeted therapeutics.

Providing a broader perspective, the reviews included in this Special Issue titled “The Role of Cryoprotective Agents in Liposome Stabilization and Preservation” and “Antifreeze Proteins: Novel Applications and Navigation towards Their Clinical Application in Cryobanking” highlighted how understanding the biophysical properties of cryoprotectants and anti-freeze proteins can improve the preservation of biological materials. The review on cryoprotective agents emphasized the importance of understanding the complex interactions between protectants and bilayer composition, which could lead to enhanced stability of liposomal drug delivery systems. The review on anti-freeze proteins emphasized their potential in the cryopreservation of different cells, tissues, and organs, pointing towards a promising future with less toxicity and more efficiency.

In essence, this Special Issue has expanded our understanding of cellular biophysics, particularly regarding membrane dynamics and the fascinating world of cryoprotection. Yet, much more remains to be explored. A deeper comprehension of lipid rafts and their manipulation could provide a plethora of therapeutic possibilities. Moreover, the search for novel cryoprotectants and their potential applications continues to be a thrilling domain of research. We must also strive to integrate these biophysical findings with cellular biology, biochemistry, and genetics, fostering a multi-disciplinary approach.

In conclusion, as the frontier of molecular and membrane biophysics continues to evolve, we look forward to more groundbreaking research that would provide insights into the delicate dance of life within the cell, ultimately leading to innovative therapeutic strategies and technological advancements in the field of cellular biology.
